# DMP1-Mediated FAK Activation Contributes to P Utilization of Broiler Osteoblasts by Suppressing FGF23 Expression

**DOI:** 10.3390/biology15020121

**Published:** 2026-01-08

**Authors:** Tingting Li, Xinyu Feng, Weiyun Zhang, Jingyi Zhao, Liyang Zhang, Yun Hu, Xiaoyan Cui, Shengchen Wang, Xugang Luo

**Affiliations:** 1 Poultry Mineral Nutrition Laboratory, College of Animal Science and Technology, Yangzhou University, Yangzhou 225012, China; 2 Mineral Nutrition Research Division, Institute of Animal Science, Chinese Academy of Agricultural Sciences, Beijing 100193, China

**Keywords:** broiler, DMP1, FAK, FGF23, P utilization, tibia osteoblasts

## Abstract

Improving phosphorus (P) utilization in broilers is crucial for reducing feed costs and environmental pollution. Bone mineralization trait is strongly associated with P utilization in poultry and is thus often used as an alternative trait for evaluating P utilization. Dentin matrix protein 1 (DMP1), an essential matrix protein for bone mineralization and P deposition, has been shown to be actively involved in P utilization in broilers, but the underlying mechanisms remain unclear. The current study aimed to investigate the possible mechanisms whereby DMP1 regulates P utilization of poultry by using gene silencing and overexpression technologies, combined with an in vitro model of primary broiler osteoblasts. The results showed that DMP1 overexpression augmented the P utilization of broiler osteoblasts, characterized by significant increases (*p* < 0.001) in P utilization rate, mineralization formation, alkaline phosphatase activity, and bone gla protein content. Meanwhile, DMP1 overexpression effectively (*p* < 0.05) activated the focal adhesion kinase (FAK) signaling, along with obvious (*p* < 0.01) decreases in fibroblast growth factor 23 (FGF23) expression and production. In contrast, DMP1 silencing reversed (*p* < 0.05) the above effects. Consistently, FAK activation promoted (*p* < 0.05) P utilization accompanied by remarkable (*p* < 0.05) decreases in FGF23 expression and production. Furthermore, gain- and loss-of-function assays demonstrated that a high level of FGF23 contributed to impaired P utilization, while a low level was beneficial. Interestingly, blocking FAK signaling not only recovered (*p* < 0.05) the FGF23 expression and production in DMP1 overexpressed cells but also obviously (*p* < 0.05) weakened their P utilization. These findings indicate that DMP1 inhibits FGF23 expression by activating FAK, thereby contributing to P utilization in broiler osteoblasts. They reveal a novel DMP1-FAK-FGF23 regulatory axis in broiler osteoblasts and provide a potential target for improving P efficiency in poultry.

## Simple Summary

This study was conducted to explore how dentin matrix protein 1 (DMP1) promotes phosphorus (P) utilization of broiler osteoblasts. Our results reveal that suppressing fibroblast growth factor 23 (FGF23) expression is beneficial for P utilization of broiler osteoblasts. Interestingly, DMP1 inhibits FGF23 expression in broiler osteoblasts, which appears to be mediated through the focal adhesion kinase (FAK) activation. This process allows the broiler osteoblasts to use P more efficiently. Our results offer a novel insight into the functionality of DMP1, which would be useful for formulating feasible strategies and/or breeding programs to improve P utilization in poultry.

## 1. Introduction

Phosphorus (P) is an essential nutrient for life, yet a costly feed ingredient, in animal diets, whose excreted form can even become an environmental pollutant if runoff carries it into surface waters [1,2]. Therefore, means to reduce P supplementation and enhance its utilization in animals are crucial, not only for reducing feed costs but also for minimizing environmental impact. However, current investigations primarily focus on how to enhance the digestibility of dietary P sources in poultry diets [3,4,5,6,7], while relatively little attention has been given to improving P utilization and its underlying mechanisms.

Dentin matrix protein 1 (DMP1) is critical for hydroxyapatite formation in vitro [8,9] and bone mineralization in vivo [10]. Studies have shown that DMP1 overexpression accelerated bone mineralization of mice [11], whereas DMP1-deleted mice showed evident osteomalacia and reduced mineral deposition [12]. In poultry, bone mineralization is an alternative trait for evaluating P utilization because of its strong correlation with P utilization [13,14,15,16]. Our recent study indicated that DMP1 expression was positively correlated with P deposition and bone mineralization related parameters in broiler tibia [17], suggesting its potential role in contributing to P utilization in broilers. Similar results were also found by in vitro experiments [18], where DMP1 expression was positively associated with the number and area of mineralized nodules in broiler tibia osteoblasts. Furthermore, DMP1 silencing exhibited obvious inhibitory effects on the P utilization rate, mineralization formation, alkaline phosphatase (ALP) activity, and bone gla protein (BGP) content in broiler tibia osteoblasts [19], further indicating its local and direct effect on bone P utilization. Nevertheless, the mechanisms underlying this effect remain unclear. As a regulator of P homeostasis, lower fibroblast growth factor 23 (FGF23) was discovered to be beneficial for promoting P absorption in laying hens fed P-restricted diets [20]. Chicks or laying hens immunized with anti-FGF23 antibody showed higher bone P retention and lower P excretion compared to non-immunized birds [21,22,23]. These findings suggest that suppressing FGF23 is an effective strategy to enhance the P utilization of poultry. Interestingly, DMP1 knockout always comes with high FGF23 level in mammals [24,25]. Moreover, exogenous DMP1 activated focal adhesion kinase (FAK) signaling [26,27], while blocking FAK significantly impaired the inhibitory effect of DMP1 stimulation on the FGF23 expression in UMR-106 or MC3T3-E1 osteoblastic cells [26]. Based on the above findings, we hypothesize that DMP1 inhibits FGF23 expression by activating FAK, thereby enhancing P utilization in broiler tibia osteoblasts. The objective of this study was to investigate the functional role of DMP1 in P utilization and its underlying molecular mechanisms by using gene silencing and overexpression technologies, combined with an in vitro model of primary broiler osteoblasts [18,19,28].

## 2. Materials and Methods

### 2.1. Ethics Statement

The animal experiments were approved by the Animal Care Committee of Yangzhou University (Permit No. SYXK (Su) 2021-0027). Every effort was taken to minimize animal suffering.

### 2.2. Broiler Primary Osteoblasts Isolation and Culture

Broiler primary osteoblasts were isolated and cultivated as our previous method [18]. Briefly, the tibial cortical bones of 1-day-old male broiler chicks were separated, split longitudinally, and then scraped plus cleaned to completely remove their connective tissues, periosteum and bone marrow. Afterwards, the cleaned bones were cut into small pieces, and then plated in 60 mm dishes with a complete culture medium consisting of low-glucose DMEM (Macgene, Beijing, China) supplemented with 15% fetal bovine serum (Gibco, Auckland, New Zealand), 1% penicillin–streptomycin and 1% L-glutamine (Gibco, Grand Island, NY, USA). Once the cells migrated from bone particles and reached confluence, the cells were digested with 0.25% Trypsin-EDTA (Macgene, Beijing, China), subcultured in rat-tail collagen-coated 6-well plates (5 × 10^5^ cells/well) and cultured with the complete culture medium. These subcultured cells were marked as passage one (P1). All cells used for subsequent experiments were at P1.

### 2.3. Small Interference RNA (siRNA) Synthesis, Plasmid Construction and Cell Transfection

The siRNAs targeting *DMP1*, *FAK* and *FGF23*, as well as negative control siRNA (si-NC), were synthesized by GenePharma Co., Ltd. (Shanghai, China), and their sequences were listed in Table 1. To generate overexpression plasmids, the coding sequence regions from *DMP1*, *FAK* or *FGF23* were cloned into pcDNA3.1-3×FLAG vector (GenScript, Wuhan, China), designated as pcDNA3.1-DMP1, pcDNA3.1-FAK and pcDNA3.1-FGF23, respectively. The vector pcDNA3.1-NC was used as a negative control. When the P1 osteoblasts grew to 60–80% confluence, siRNA (80 pmol/well) or plasmids (1 μg/well) were transfected using jetPRIME^®^ Transfection Reagent (Polyplus, Illkirch-Graffenstaden, France). After 24 h, the cells were washed with the P-free medium (DMEM with no phosphate; Macgene, Beijing, China), and then incubated in osteogenic medium as described previously [19,29] for 7 days. The osteogenic medium was the complete culture medium supplemented with 100 nmol/L dexamethasone, 50 μg/mL ascorbic acid and 10 mmol/L sodium β-glycerophosphate. The transfection was repeated once after 3 days of incubation in osteogenic medium [19].

### 2.4. Quantitative Real-Time PCR (RT-qPCR)

After 72 h of the first transfection, the cells were collected to extract total RNA using the TRIzol reagent (Solarbio, Beijing, China). Reverse transcription was performed using the HiScript II Q RT SuperMix kit (Vazyme, Nanjing, China) according to the manufacturer’s manual. The specific primers for *DMP1*, *FAK*, *FGF23*, *β-actin*, and *GAPDH* were designed and presented in Table 1. The RT-qPCR was carried out with the SupRealQ Purple Universal SYBR qPCR Master Mix (Vazyme, Nanjing, China) on an ABI QuantStudio 3 System (Applied Biosystem Inc., Foster City, CA, USA). Gene expression levels were normalized to the geometric mean of the two housekeeping genes (*GAPDH* and *β-actin*) using the 2^−ΔΔCt^ method.

### 2.5. Western Blot Analysis

After 72 h of the first transfection, the cells were lysed in RIPA buffer (Beyotime, Shanghai, China) for 10 min on ice to extract cellular protein. The total protein concentration was determined using a BCA Kit (Pierce, Rockford, IL, USA). Subsequently, protein samples (10 μg) were separated on a 10% SDS-PAGE gel and transferred onto a nitrocellulose membrane (Merck Millipore, Burlington, VT, USA). After being blocked in a 5% BSA or skim milk solution for 1 h at room temperature, the membranes were incubated overnight at 4 °C with primary antibodies against DMP1 (at 1:500 dilution; Abclonal, Wuhan, China), FLAG (at 1:5000 dilution; Mabnus, Wuhan, China), FAK (at 1: 500 dilution; BD Biosciences, San Jose, CA, USA), phosphorylated (p-) FAK (at 1:1000 dilution; Abcam, Cambridge, UK), FGF23 (at 1:500 dilution; Abclonal, Wuhan, China), and β-actin (at 1:5000 dilution; Huaxingbio, Beijing, China), followed by the incubation with goat anti-rabbit or anti-mouse secondary antibody (at 1:5000 dilution; Huaxingbio, Beijing, China). Finally, the membranes were developed in an enhanced chemiluminescence solution (Tanon, Shanghai, China) and then imaged using a Tanon 5200 luminometer (Shanghai Tanon Co., Ltd., Shanghai, China), followed by analysis with its own GIS software (4.2 version). β-actin was used as an internal standard to normalize the targeted protein levels.

### 2.6. Determination of ALP Activity, BGP and FGF23 Contents in Osteoblasts

Five days after the osteogenic incubation, cells were scraped into PBS (0.3 mL/well) and lysed with ultrasonication at a power of 300 W (5 s × 4 times). Subsequently, the cell lysates were centrifuged, and the supernatant was collected for the analysis of ALP activity, as well as the contents of BGP and FGF23. The ALP activity was measured using a microplate reader along with ALP assay kits (Jiancheng, Nanjing, China). The contents of BGP and FGF23 were determined by ELISA kits (Jiancheng, Nanjing, China). All three were normalized with total protein concentration of each sample.

### 2.7. Detection and Quantification of Mineralization Formation

Detection and quantification of mineralization were determined as described previously [30]. Seven days after the osteogenic incubation, the cells were first fixed with 4% paraformaldehyde for 15 min at room temperature. After washing with PBS, the fixed cells were stained with 1% Alizarin Red S (ARS, pH 4.2; Solarbio, Beijing, China) for 20 min, and then rinsed with PBS another three times. To quantify the mineralization staining, the stained cells were incubated with 10% (*v*/*v*) acetic acid (800 µL/well) for 30 min on a shaker. Then, the slurry from each well was transferred into a 1.5 mL tube and vortexed for 30 s. Next, the tubes were heated at 85 °C for 10 min, chilled, and centrifuged at 20,000× *g* for 15 min. 500 µL of supernatant from each tube was taken to mix with 200 µL of 10% (*v*/*v*) ammonium hydroxide. Finally, the absorbance of the mixture (150 µL, pH 4.2) was read at 405 nm in 96-well with opaque wall and transparent bottom. Standard curve of ARS was prepared to calculate the released ARS concentration. In the present study, the mineralization formation was represented by the ARS concentration [19,31].

### 2.8. Phosphorus Utilization Rate

The P utilization rate of broiler osteoblasts was determined according to the method described previously [18,19]. Briefly, the old medium from each well was collected and pooled at every medium replacement. The total P contents in fresh or old medium was determined by 5110 plasma emission spectrometer (5110 ICP-OES, Agilent Technologies Australia (M) Pty. Ltd, Melbourne, VIC, Australia). The P utilization rate of broiler osteoblasts was calculated as follows:

(1) $$P\;\mathrm{utilization}\;\mathrm{rate}\;\left(\%\right)=\;\frac{V1\;\times \;C1\;-\;V2\;\times \;C2}{V1\;\times \;C1}\;\times \;100$$

where V1 and V2 represent the total volume (mL) of the added fresh medium and the pooled old medium, respectively. C1 and C2 represent the total P content (mmol/L) in the fresh medium and the old medium, respectively.

### 2.9. Statistical Analyses

Statistical analyses were performed by *t*-test or one-way ANOVA with LSD post hoc test using the GLM procedure of SAS 9.4 (SAS Institute Inc., Cary, NC, USA). The replicate served as the experimental unit. Statistical significance was set at *p* < 0.05.

## 3. Results

### 3.1. Regulation Role of DMP1 in FAK and FGF23 Expression of Broiler Osteoblasts

**Figure 1:**
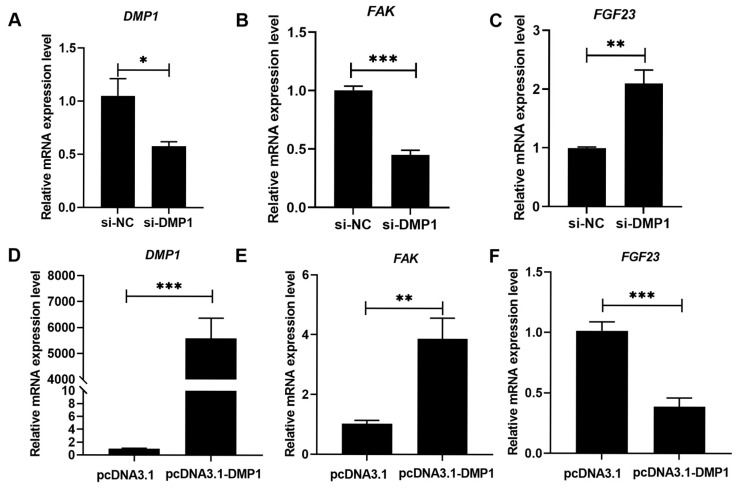
The effect of si-DMP1 or pcDNA3.1-DMP1 transfection on the mRNA expression levels of *DMP1* (**A**,**D**), *FAK* (**B**,**E**), and *FGF23* (**C**,**F**) in broiler osteoblasts. All values are expressed as means ± SE, *n* = 5–6. * *p* < 0.05, ** *p* < 0.01, *** *p* < 0.001. DMP1, dentin matrix protein 1; FAK, focal adhesion kinase; FGF23, fibroblast growth factor 23.

**Figure 2:**
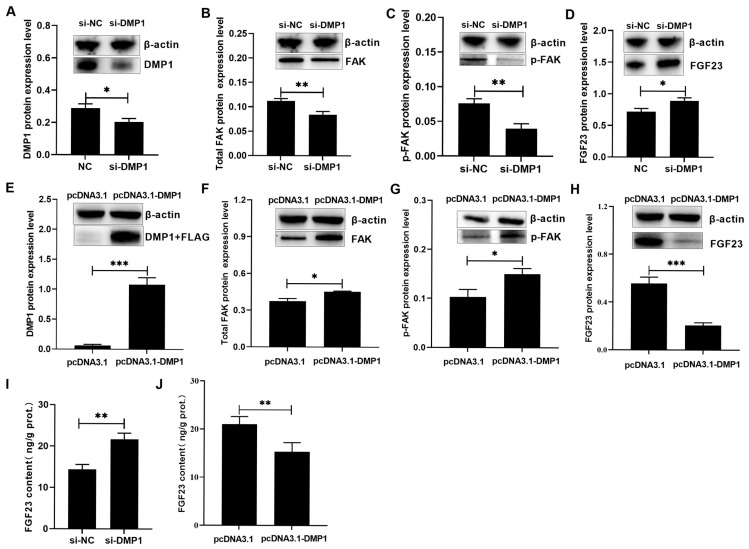
The effect of si-DMP1 or pcDNA3.1-DMP1 transfection on the protein expression levels of DMP1 (**A**,**E**), FAK (**B**,**F**), p-FAK (**C**,**G**), FGF23 (**D**,**H**), and FGF23 content (**I**,**J**) in broiler osteoblasts. All values are expressed as means ± SE, *n* = 4–6. * *p* < 0.05, ** *p* < 0.01, *** *p* < 0.001. DMP1, dentin matrix protein 1; FAK, focal adhesion kinase; p-FAK, phosphorylated FAK; FGF23, fibroblast growth factor 23.

The possible role of DMP1 in regulating the osteoblastic FAK and FGF23 expression was presented in Figure 1 and Figure 2 (Figures S1 and S2). Compared to si-NC or pcDNA3.1, si-DMP1 or pcDNA3.1-DMP1 specifically acted to markedly (*p* < 0.05) decrease or increase the DMP1 expression in broiler osteoblasts (Figure 1A,D and Figure 2A,E). DMP1 silencing significantly (*p* < 0.01) downregulated *FAK* mRNA expression but upregulated *FGF23* mRNA expression (Figure 1B,C). On the contrary, DMP1 overexpression had the opposite effect (*p* < 0.01) on their mRNA expression (Figure 1E,F). Furthermore, DMP1 silencing decreased (*p* < 0.01) the total and phosphorylated FAK protein levels (Figure 2B,C), along with obvious increases (*p* < 0.05) in FGF23 protein abundance and production in broiler osteoblasts (Figure 2D,I). However, DMP1 overexpression elevated (*p* < 0.05) the total and phosphorylated FAK protein levels (Figure 2F,G), accompanied by obvious decreases (*p* < 0.01) in FGF23 protein abundance and production in broiler osteoblasts (Figure 2H,J).

### 3.2. DMP1 Is a Positive Regulator for P Utilization of Broiler Osteoblasts

**Figure 3:**
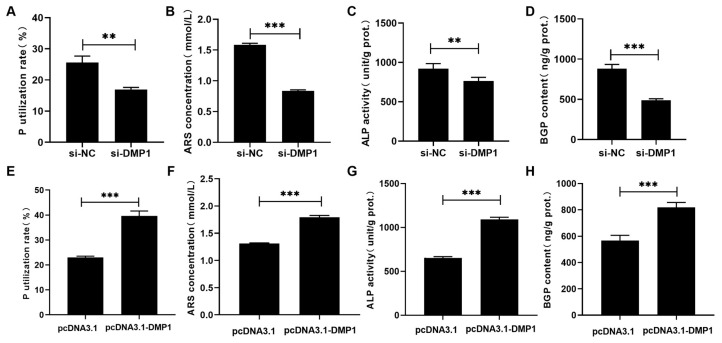
The effects of DMP1 silencing and overexpression on the P utilization rate (**A**,**E**), ARS concentration (**B**,**F**), ALP activity (**C**,**G**) and BGP content (**D**,**H**) in broiler osteoblasts. All values are expressed as means ± SE, *n* = 6. ** *p* < 0.01, *** *p* < 0.001. DMP1, dentin matrix protein 1; ARS, alizarin red S; ALP, alkaline phosphatase activity; BGP, bone gla protein. ARS concentration (mmol/L) was used to represent the mineralization formation.

Effects of DMP1 silencing and overexpression on the P utilization of broiler osteoblasts are shown in Figure 3. DMP1 silencing significantly (*p* < 0.01) decreased the P utilization rate, mineralization formation, ALP activity, and BGP content in osteoblasts (Figure 3A–D). In contrast, DMP1 overexpression evidently (*p* < 0.001) augmented the above P utilization related parameters (Figure 3E–H).

### 3.3. High FGF23 Level Contributes to Impaired P Utilization in Broiler Osteoblasts

**Figure 4:**
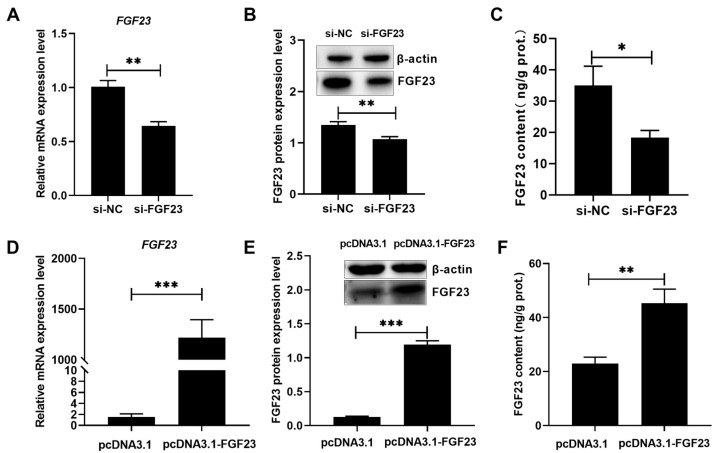
The effect of si-FGF23 or pcDNA3.1-FGF23 transfection on *FGF23* mRNA expression (**A**,**D**), FGF23 protein expression (**B**,**E**), and FGF23 content (**C**,**F**) in broiler osteoblasts. All values are expressed as means ± SE, *n* = 6. * *p* < 0.05, ** *p* < 0.01, *** *p* < 0.001. FGF23, fibroblast growth factor 23.

**Figure 5:**
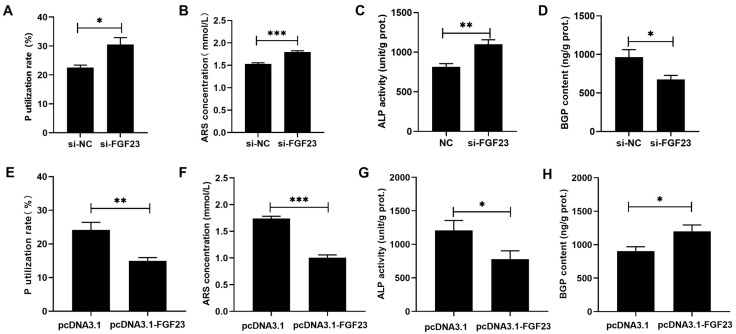
The effects of FGF23 silencing and overexpression on the P utilization rate (**A**,**E**), ARS concentration (**B**,**F**), ALP activity (**C**,**G**) and BGP content (**D**,**H**) in broiler osteoblasts. All values are expressed as means ± SE, *n* = 6. * *p* < 0.05, ** *p* < 0.01, *** *p* < 0.001. FGF23, fibroblast growth factor 23; ARS, alizarin red S; ALP, alkaline phosphatase activity; BGP, bone gla protein. ARS concentration (mmol/L) was used to represent the mineralization formation.

According to the results in Figure 4 and Figure S3, si-FGF23 and pcDNA3.1-FGF23 could effectively (*p* < 0.05) reduce and increase the levels of *FGF23* mRNA, protein, and production in broiler osteoblasts. FGF23 silencing significantly (*p* < 0.05) augmented the P utilization rate, mineralization formation, ALP activity, but reduced the BGP content in broiler osteoblasts (Figure 5A–D), while FGF23 overexpression acted reverse effects (Figure 5E–H).

### 3.4. FAK Activation Enhanced P Utilization but Inhibited FGF23 Expression

**Figure 6:**
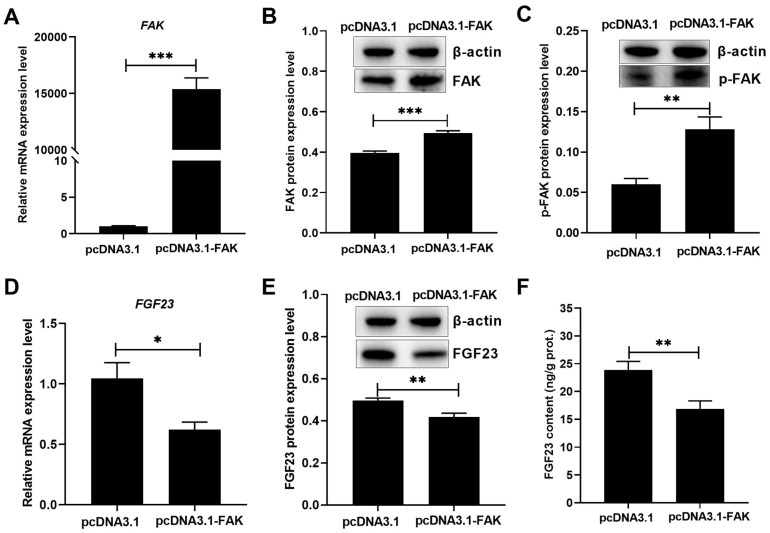
FAK overexpression decreased FGF23 expression and production in broiler osteoblasts. (**A**) *FAK* mRNA. (**B**) Total FAK protein. (**C**) p-FAK protein. (**D**) FGF23 mRNA. (**E**) FGF23 protein. (**F**) FGF23 content. All values are expressed as means ± SE, *n* = 4–6. * *p* < 0.05, ** *p* < 0.01, *** *p* < 0.001. FAK, focal adhesion kinase; p-FAK, phosphorylated FAK; FGF23, fibroblast growth factor 23.

**Figure 7:**
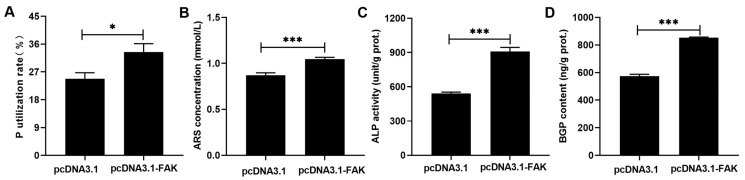
FAK overexpression augmented the P utilization rate (**A**), ARS concentration (**B**), ALP activity (**C**) and BGP content (**D**) in broiler osteoblasts. All values are expressed as means ± SE, *n* = 6. * *p* < 0.05, *** *p* < 0.001. FAK, focal adhesion kinase; ARS, alizarin red S; ALP, alkaline phosphatase activity; BGP, bone gla protein. ARS concentration (mmol/L) was used to represent the mineralization formation.

The pcDNA3.1-FAK transfection markedly (*p* < 0.01) increased the *FAK* mRNA expression level (Figure 6A), total and phosphorylated FAK protein levels (Figure 6B,C and Figure S4) in broiler osteoblasts. FAK overexpression evidently (*p* < 0.05) elevated the P utilization rate, mineralization formation, ALP activity, and BGP content in broiler osteoblasts (Figure 7A–D), accompanied with significant (*p* < 0.05) decreases in the levels of *FGF23* mRNA (Figure 6D), protein (Figure 6E and Figure S4), and production (Figure 6F) in broiler osteoblasts.

### 3.5. Attenuated FAK Weakened the Inhibitory Effect of DMP1 on FGF23 Expression in Broiler Osteoblasts

**Figure 8:**
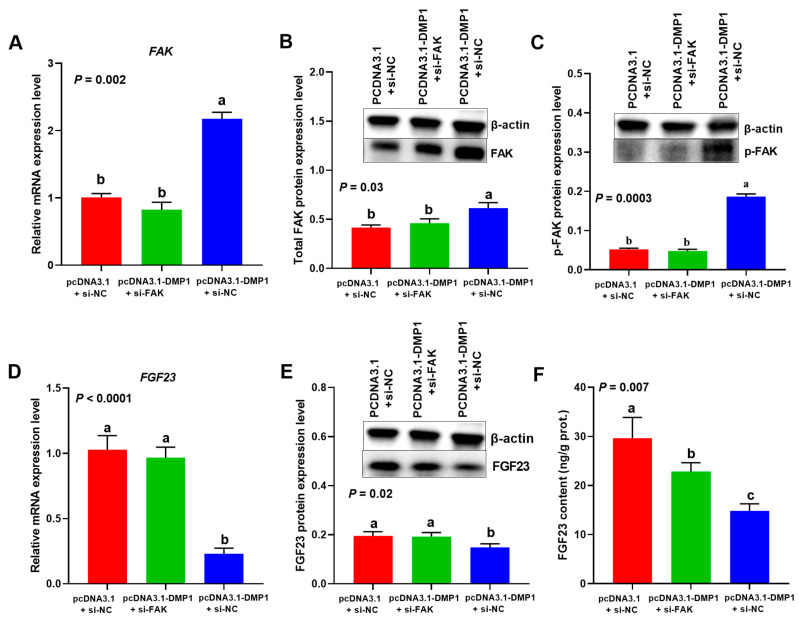
Attenuated FAK weakened the inhibitory effect of DMP1 on FGF23 expression in broiler osteoblasts. (**A**) *FAK* mRNA. (**B**) Total FAK protein. (**C**) p-FAK protein. (**D**) FGF23 mRNA. (**E**) FGF23 protein. (**F**) FGF23 content. All values are expressed as means ± SE, *n* = 3–6. Lacking the same letters (a–c) means differences at *p* < 0.05. DMP1, dentin matrix protein 1; FAK, focal adhesion kinase; p-FAK, phosphorylated FAK; FGF23, fibroblast growth factor 23.

Compared with the control group (cells transfected with si-NC and pcDNA3.1), DMP1 overexpression (cells transfected with si-NC and pcDNA3.1-DMP1) significantly (*p* < 0.05) upregulated the expression levels of *FAK* mRNA (Figure 8A), total and phosphorylated FAK protein (Figure 8B,C and Figure S5), but decreased the levels of *FGF23* mRNA (Figure 8D), protein (Figure 8E and Figure S5), and production (Figure 8F) in broiler osteoblasts. Interestingly, the decreased FGF23 expression and production were markedly (*p* < 0.05) recovered by co-transfection of si-FAK, a siRNA for specifically inhibiting FAK expression (Figure 8D–F).

### 3.6. Attenuated FAK Impaired the Promotive Effect of DMP1 on P Utilization in Broiler Osteoblasts

**Figure 9:**
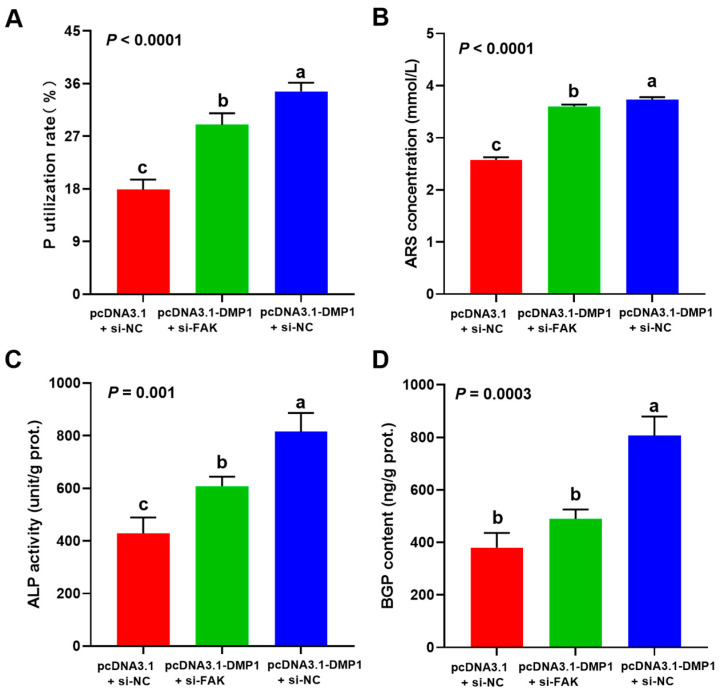
Attenuated FAK impaired the promotive effect of DMP1 the P utilization rate (**A**), ARS concentration (**B**), ALP activity (**C**) and BGP content (**D**) in broiler osteoblasts. All values are expressed as means ± SE, *n* = 6. Lacking the same letters (a–c) means differences at *p* < 0.05. DMP1, dentin matrix protein 1; FAK, focal adhesion kinase; ARS, alizarin red S; ALP, alkaline phosphatase activity; BGP, bone gla protein. ARS concentration (mmol/L) was used to represent the mineralization formation.

DMP1 overexpression significantly (*p* < 0.05) increased the P utilization rate, mineralization formation, ALP activity, and BGP content in broiler osteoblasts, while these effects were evidently (*p* < 0.05) weakened when the FAK signaling was blocking by si-FAK (Figure 9A–D).

## 4. Discussion

In poultry, bone mineralization and P deposition can reflect the utilization efficiency of P in the body to a large extent. Consequently, improving the bone P utilization and comprehending its underlying mechanisms are essential for formulating viable strategies to optimize dietary P requirements and minimize environmental P excretion in the poultry industry. The hypothesis that DMP1 inhibits FGF23 expression by activating FAK, thereby enhancing P utilization in broiler osteoblasts has been supported by the results of the present study. We find that DMP1 and FAK are positive regulators for P utilization of broiler osteoblasts, whereas FGF23 is a negative regulator. Mechanistically, FAK activation is required for DMP1 to exert its inhibitory effect on FGF23 expression. Blocking FAK signaling not only recovered the FGF23 expression and production in DMP1-overexpressed cells, but also obviously weakened their P utilization. These findings provide novel insights into bone P utilization and its mechanisms in broilers, and will contribute to progress in improving P utilization of poultry in the future.

It has been shown that DMP1 is essential for regulating P homeostasis in organisms [10,32]. In mammals, the deletion or mutation of DMP1 leads to hypophosphatemia, bone mineralization defects, and excessive P excretion in urine [12,24,33], while overexpressing DMP1 accelerates bone mineralization with high bone mineral density [11]. Similarly, the present study found that DMP1 overexpression enhanced the P utilization of broiler osteoblasts, characterized by obvious increases in the P utilization rate, mineralization formation, ALP activity, and BGP content. In contrast, DMP1 silencing had the opposite effect, which was consistent with our previous study [19]. Collectively, these findings suggest that DMP1 functions as a positive regulator of P utilization. Numerous in vivo studies have shown the deficiency of DMP1 is always accompanied by high circulating FGF23 [34,35,36], a P-regulating hormone synthesized by osteoblasts. Excessive FGF23 inhibits the intestinal P absorption and renal P resorption [37,38,39,40], thereby decreasing the utilization efficiency of P. Conversely, FGF23 neutralization not only reduced the P requirement of growing broiler chicks but also decreased the P excretion of laying hens [20,22,23,41]. Besides serving as a systemic hormone, FGF23 also acts as local bone-derived factor to directly regulating bone mineralization. Lyu et al. and Wang et al. reported that FG23 overexpression suppressed matrix mineralization and ALP activity in osteoblasts-like cells [42,43]. This is in accordance with our results, where FGF23 overexpression inhibited the P utilization rate, mineralization formation, and ALP activity, but FGF23 silencing reversed them. It follows that whether acting as a systemic hormone or a local bone-derived factor, FGF23 is a negative regulator of P utilization in poultry. Interestingly, the FGF23 expression and production of broiler osteoblasts were negatively regulated by DMP1 in the present study. Moreover, the correction of FGF23 excess, either by blocking its signaling or by reducing its expression, can fully correct hypophosphatemia in DMP1 knockout mice [44,45]. Together, these findings support that DMP1 contributes to enhance P utilization by suppressing FGF23. As an extracellular matrix, DMP1 has been established to trigger the formation of focal adhesion points through the actions of the cell surface receptor (αVβ3 integrin) [27,46]; thus, we also assayed the effect of DMP1 in regulating FAK expression. Consistently, DMP1 overexpression stimulated FAK activation in broiler osteoblasts. Meanwhile, FAK overexpression augmented the P utilization along with the decreases in FGF23 expression and production. Evidences exhibited that blocking FAK, either by its inhibitors or by gene knockout, obviously delayed the mineralization formation with a decrease in ALP activity in mice osteoblasts [47,48,49,50]. Moreover, Lee et al. reported that exogenous DMP1 reduced the FGF23 contents in osteoblasts or conditional mediums, and this effect was significantly recovered by using FAK inhibitors [26]. Similar results were also found in the present study, where DMP1 overexpression suppressed the FGF23 mRNA, protein and production in broiler osteoblasts, while their levels could be recovered by blocking FAK signal by using gene interference. We further determined changes in P utilization when FAK signaling was blocking in DMP1-overexpressed osteoblasts. Interestingly, the enhanced P utilization caused by DMP1 overexpression was significantly weakened due to FAK blocking. Taken together, DMP1 inhibits FGF23 expression by activating FAK, thereby enhancing P utilization in broiler osteoblasts.

## 5. Conclusions

In conclusion, DMP1 and FAK are positive regulators of P utilization in broiler osteoblasts, while FGF23 is a negative regulator. DMP1 negatively regulates FGF23 expression in broiler osteoblasts, which appears to be mediated through FAK signaling. DMP1-medicated FAK activation contributes to P utilization of broiler osteoblasts by suppressing FGF23 expression. Considering that the in vitro cellular model cannot fully mimic the in vivo environment, additional in vivo studies are needed to verify whether enhancing the DMP1-FAK-FGF23 axis can reduce dietary P requirements or improve bone P deposition under commercial feeding conditions.

## Figures and Tables

**Table 1:** Sequences of siRNAs and primers.

Item	Name	Forward (5′-3′)	Reverse (5′-3′)
siRNAs	si-DMP1	GGAUAAGGAAGAGGGUGAATT	UUCACCCUCUUCCUUAUCCTT
	si-FAK	GAUCCUACGGAGAGAUGAGTT	CUCAUCUCUCCGUAGGAUCTT
	si-FGF23	GACUGUGUGUUCAACCAAATT	UUUGGUUGAACACACAGUCTT
	si-NC	UUCUCCGAACGUGUCACGUTT	ACGUGACACGUUCGGAGAATT
Primers for RT-qPCR	*DMP1*	GCCTGACGATGATGCTCCAA	TGGATGTGCTCTCTTCGCTC
	*FGF23*	ATGCTGCTTGTGCTCTGTATC	ACTGTAAATGGTTTGGTGAGG
	*FAK*	ATTGCTGCTAGGAACGTGCT	ACCGTCGGAAGTTGATTGAC
	*β-actin*	CAGCCATCTTTCTTGGGTAT	CTGTGATCTCCTTCTGCATCC
	*GAPDH*	GCACGCCATCACTATCTT	GGACTCCACAACATACTCAG

DMP1, dentin matrix protein 1; FAK, focal adhesion kinase; FGF23, fibroblast growth factor 23; GAPDH, glyceraldehyde-3-phosphate dehydrogenase.

## Data Availability

The original contributions presented in this study are included in the article/Supplementary Material. Further inquiries can be directed to the corresponding author(s).

## Supplementary Materials

The following supporting information can be downloaded at https://www.mdpi.com/article/10.3390/biology15020121/s1. Figure S1: Western blot data for Figure 2A–D; Figure S2: Western blot data for Figure 2E–H; Figure S3: Western blot data for Figure 4B,E; Figure S4: Western blot data for Figure 6B,C,E; Figure S5: Western blot data for Figure 8B,C,E.
