# Supplementary material for: DMP1-Mediated FAK Activation Contributes to P Utilization of Broiler Osteoblasts by Suppressing FGF23 Expression
Source: Biology (Basel). 2026 Jan 8;15(2):121. doi: 10.3390/biology15020121 (PMC12838098; doi:10.3390/biology15020121)
Supplement: Supplementary file 1 [file biology-15-00121-s001.zip › biology-4075508-supplementary.pdf]

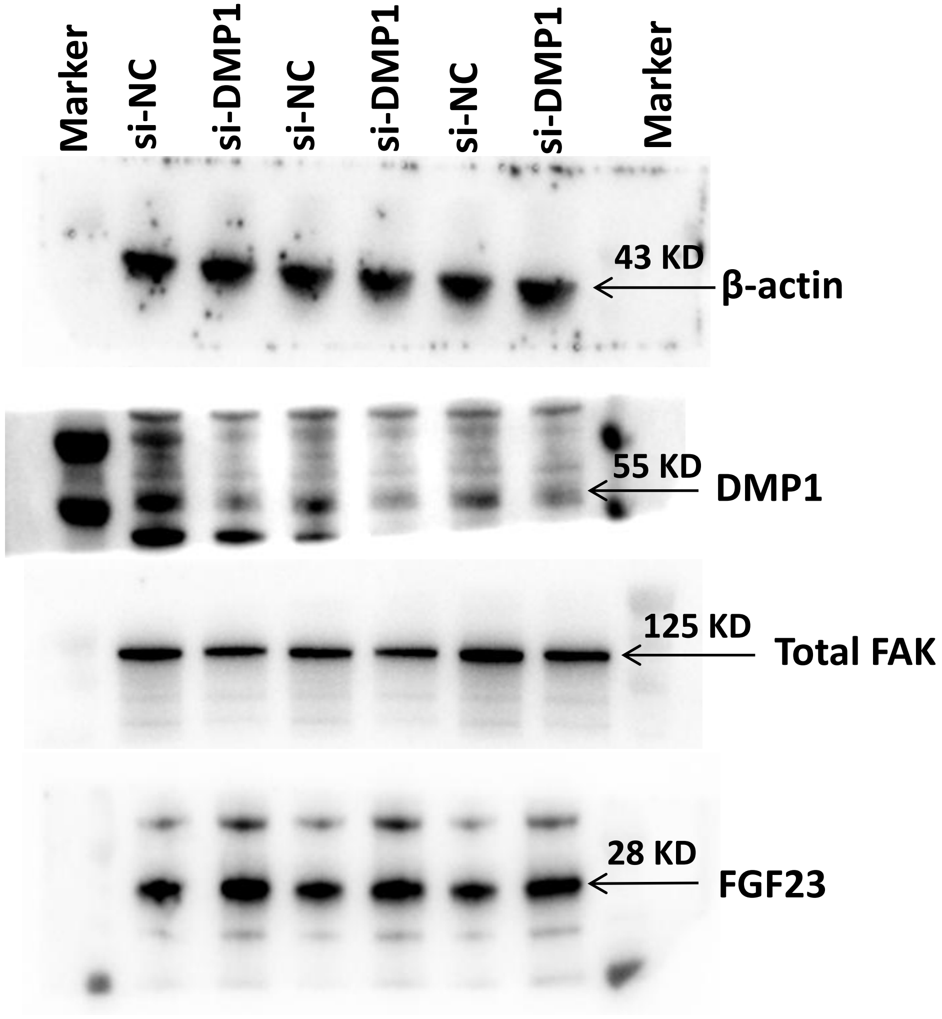

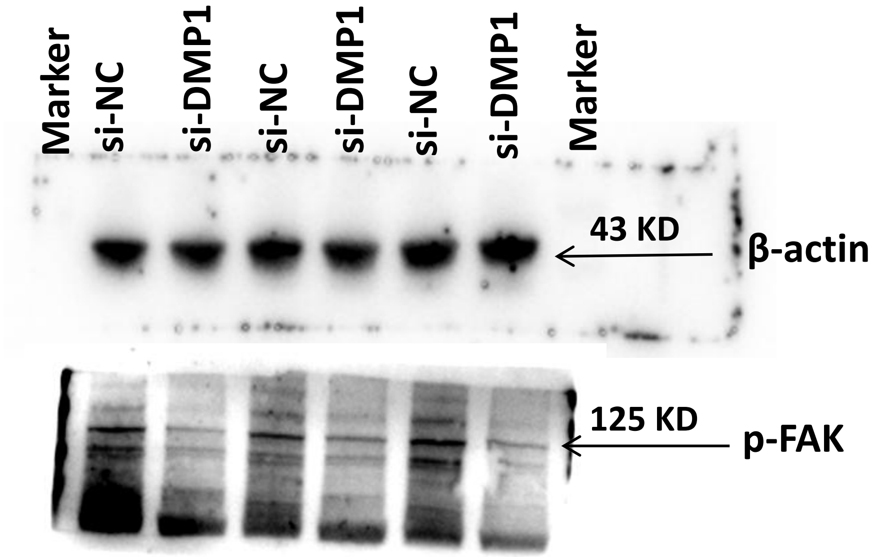

Figure S1. Western blot data for Figure 2A, B, C, and D.

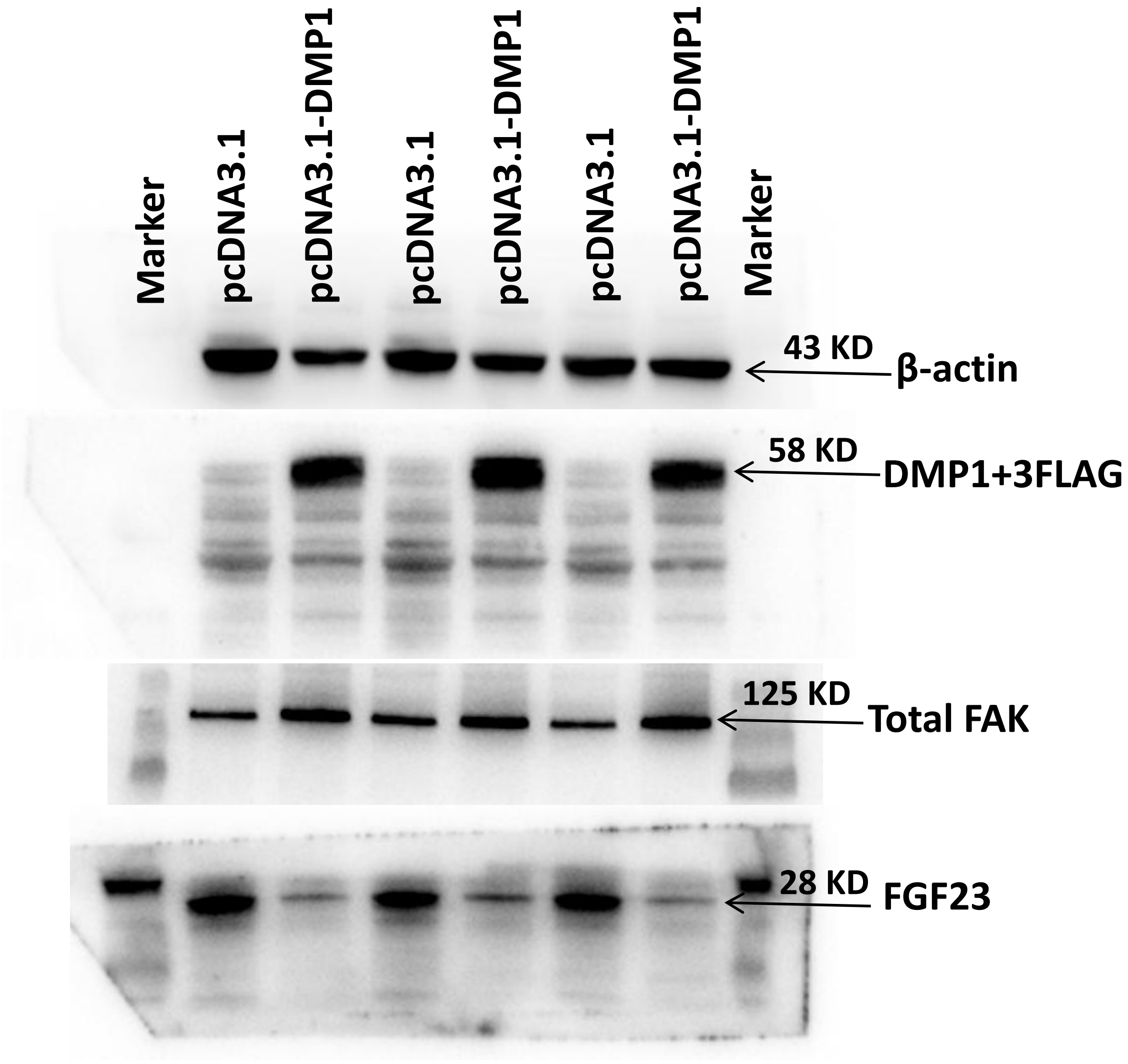

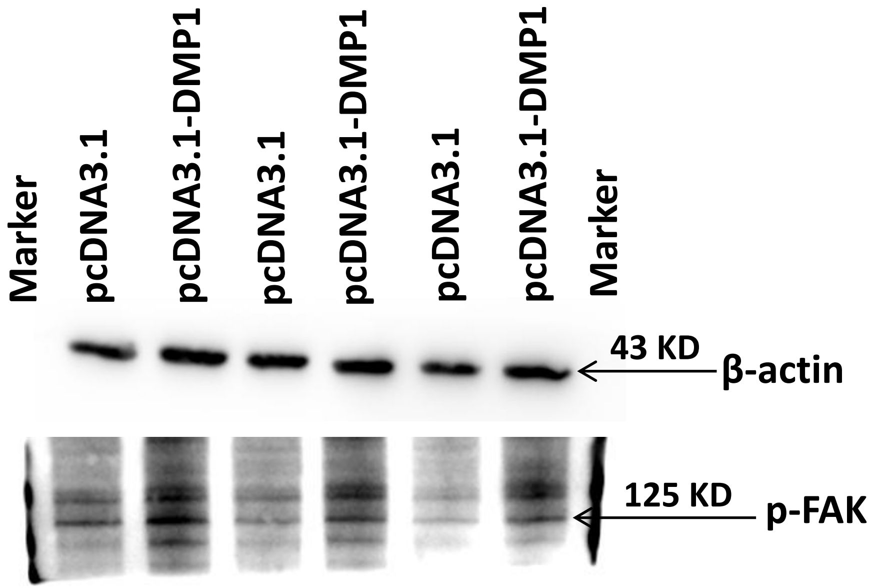

Figure S2. Western blot data for Figure 2E, F, G, and H.

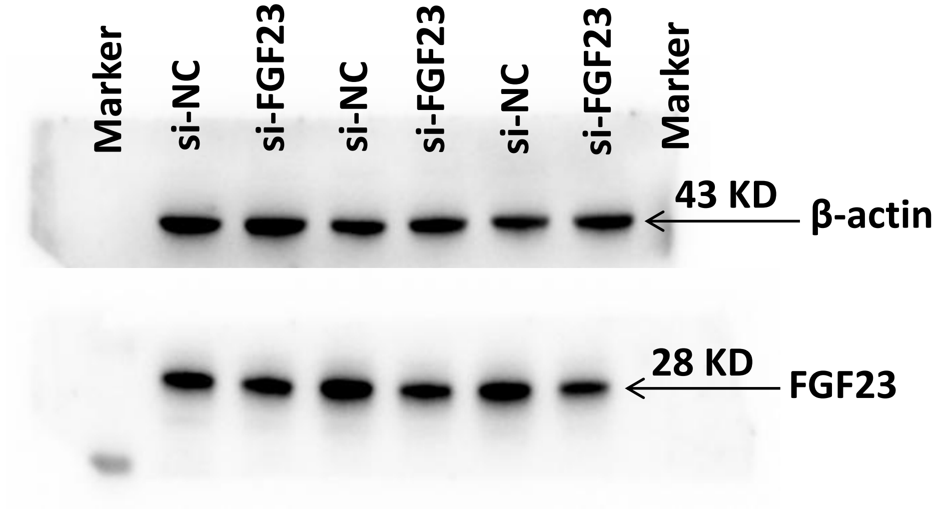

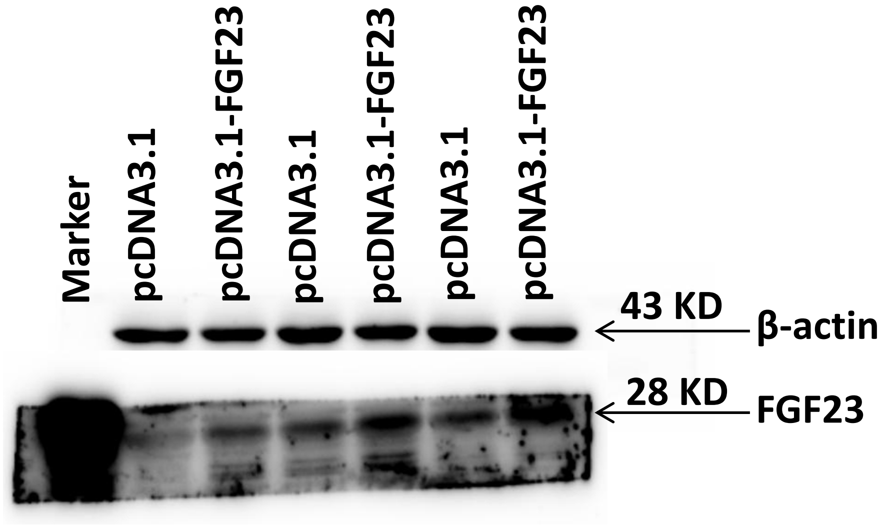

Figure S3. Western blot data for Figure 4B and E.

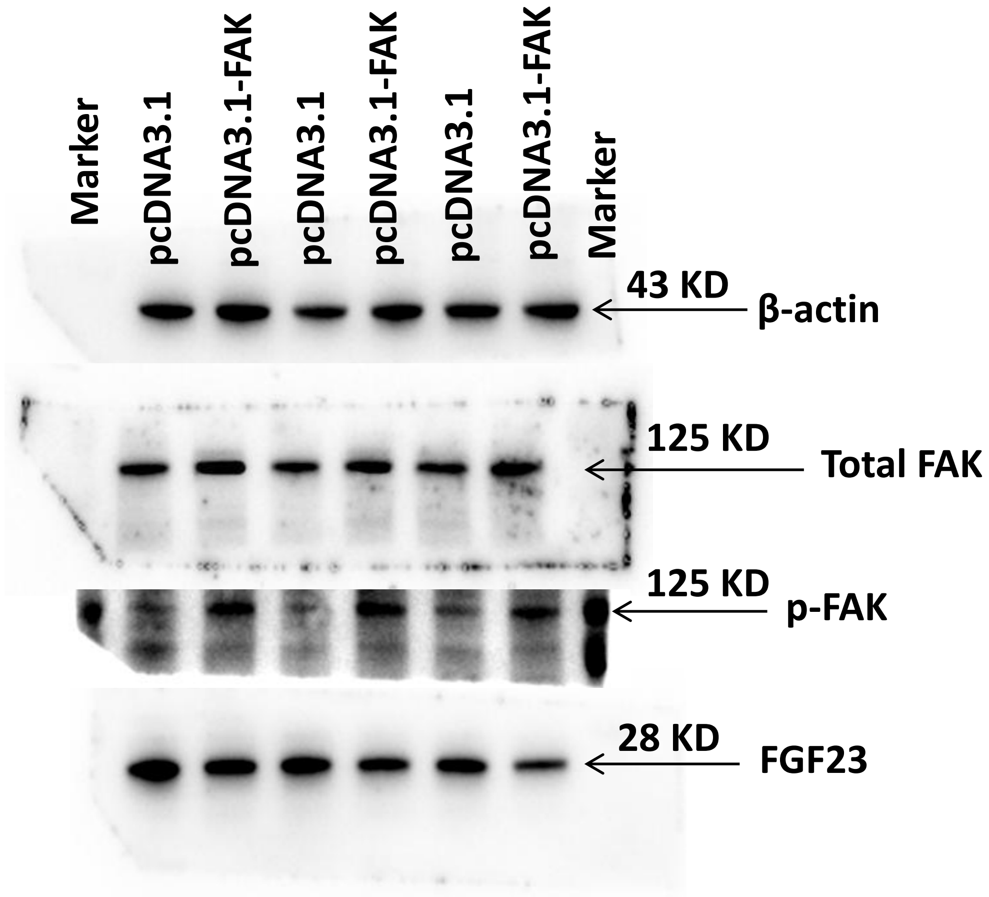

Figure S4. Western blot data for Figure 6B, C, and E.

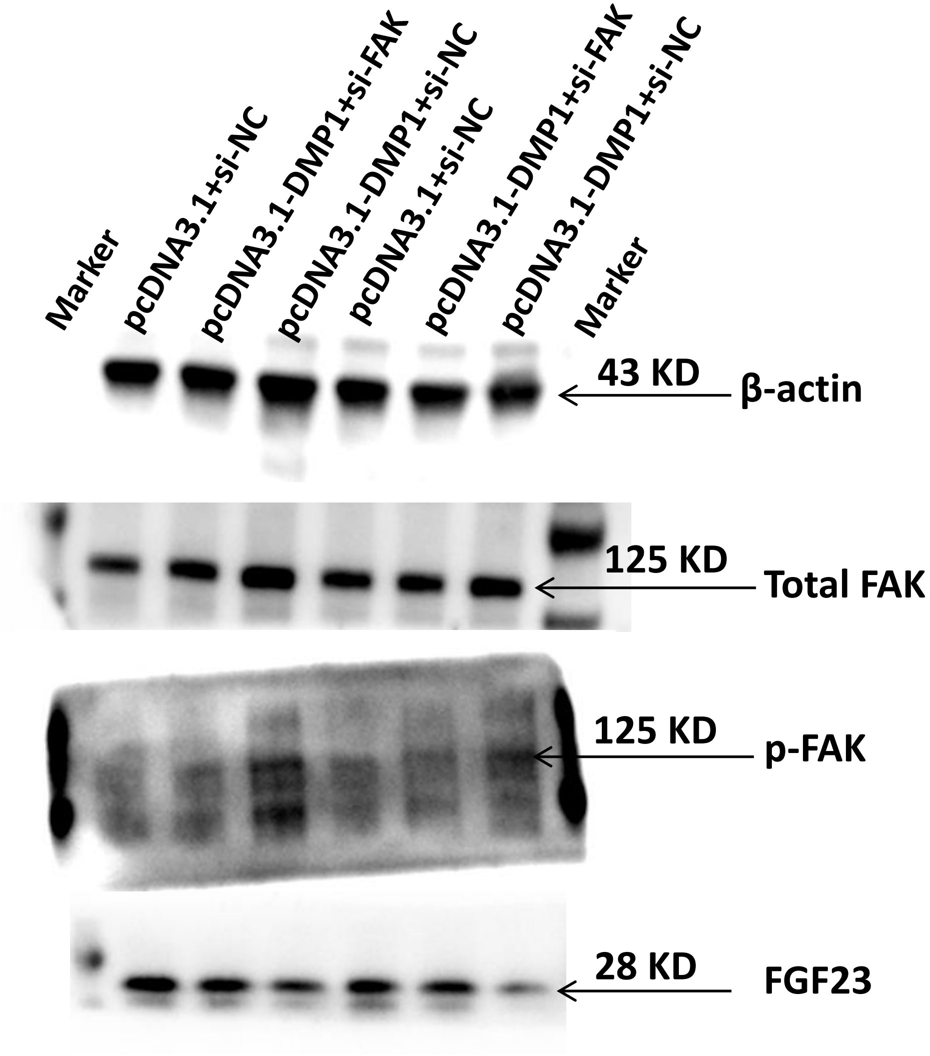

Figure S5. Western blot data for Figure 8B, C, and E.
